# A Manual of the Practice of Medicine

**Published:** 1896-09

**Authors:** 


**A Manual of the Practice of Medicine.** By George Roe Lockwood, M.D. Pp. 935. Philadelphia: W. B. Saunders. 1896.

A text-book of medicine, of good type, complete in less than a thousand octavo pages, certainly deserves to be spoken of as concise and available. The classification of Osler has been adopted, and the book is well illustrated with twenty-two full-page coloured plates and seventy-five illustrations in the text. The book is an excellent one for the student, and quite deserves its position as one of a new aid series. It is well revised and has a good index. The matter appears to be well up to date, and free from mistakes of any kind.

